# PReS-FINAL-2249: A national survey of the role of the paediatric rheumatology nurse in performing steroid joint injections in the UK

**DOI:** 10.1186/1546-0096-11-S2-P239

**Published:** 2013-12-05

**Authors:** H Lee, D Hawley, J Edgerton, M Al-Obaidi

**Affiliations:** 1 Paediatric and Adolescent Rheumatology, Sheffield Children's NHS Foundation Trust, Sheffield, UK

## Introduction

Juvenile idiopathic arthritis (JIA) is the most common rheumatic disease in children. Treatments involve Non Steroidal Anti-Inflammatory Drugs (NSAIDs), Disease Modifying Anti-Rheumatic Agents (DMARDs), steroids and various biologic drugs. Steroid injections directly into affected joints are an important part of the range of treatments available for children with JIA. The British Society of Paediatric and Adolescent Rheumatology (BSPAR) guidelines state that "all patients with JIA will have access to intra-articular joint injections as required, with access to entonox, general anaesthesia and appropriate imaging technology where necessary."

To meet the increasing demand for steroid joint injections, Rheumatology nurses and other rheumatology allied health professionals have started training to carry out this extended role in some parts of the UK.

## Objectives

To find out what has been done nationally in different Paediatric Rheumatology centres with regards to whether or not nurses are carrying out joint injections, what joints they are competent to inject, and what training they have undertaken in order to carry this role out.

## Methods

We have identified 15 Paediatric Rheumatology Centre in the country, and a questionnaire was sent out asking whether nurses do joint injections, what sort of sedation was used, whether they obtain a written consent, what joints they do/don't inject and what training did they have.

## Results

We had responses from 12 out of the 15 centres identified.

4 out of the 12 centres who replied have Rheumatology nurses performing joint injections.

In 1 out of the 4 centres the Rheumatology nurse will only carry out joint injections under general anaesthetic (GA) whereas in the 3 other centres, Rheumatology nurses are carrying out joint injections both under GA and entonox.

All 4 centres said that they would not inject hips, and 3 out of the four said that they would not inject any joint requiring imaging.

Training was varied but generally carried out by the Consultant Rheumatologist in the centre. One nurse did attend a training course but this was an adult course rather than paediatric.

Only 1 centre developed a formalised training competency pack for joint injections.

2 of the 8 other centres (currently not performing joint injections) replied to say that they were planning to start carrying out joint injections but only with entonox and 1 centre replied to say that they were looking into possible courses.

## Conclusion

The role of the Paediatric Rheumatology Nurse throughout the UK is varied and continually evolving. It would appear that the demand for steroid joint injections in different parts of the UK is a factor in determining whether or not Rheumatology Nurses take on this extended role. However, the training appears to be limited and this highlights the need for a formal training pathway.

## Disclosure of interest

None declared.

